# Detecting tuberculosis in pregnant and postpartum women in Eswatini

**DOI:** 10.4102/ajlm.v9i1.837

**Published:** 2020-07-30

**Authors:** Munyaradzi Pasipamire, Edward Broughton, Mandzisi Mkhontfo, Gugu Maphalala, Batsabile Simelane-Vilane, Samson Haumba

**Affiliations:** 1 Research and Evaluation, Eswatini National AIDS Programme, Ministry of Health, Mbabane, Eswatini; 2 Research and Evaluation, University Research Co. LLC, Chevy Chase, Maryland, United States; 3 International Health, Johns Hopkins Bloomberg School of Public Health, Baltimore, Maryland, United States; 4 University Research Co. LLC, Mbabane, Eswatini; 5 Eswatini Health Laboratory Services, Mbabane, Eswatini

**Keywords:** Tuberculosis, pregnant women, postpartum women, tuberculosis screening, tuberculosis diagnosis, HIV, Eswatini

## Abstract

**Background:**

Tuberculosis diagnosis in pregnancy is complex because tuberculosis symptoms are often masked by physiological symptoms of pregnancy. Untreated tuberculosis in pregnant and postpartum women may lead to maternal morbidity and low birth weight. Tuberculosis in HIV-positive pregnant women increases the risk of maternal and infant mortality.

**Objective:**

This study aimed to determine tuberculosis prevalence stratified by HIV status and identify screening algorithms that maximise detection of active tuberculosis among pregnant and postpartum women in Eswatini.

**Methods:**

Women were enrolled at antenatal and postnatal clinics in Eswatini for tuberculosis screening and diagnostic investigations from 01 April to 30 November 2015 in a cross-sectional study. Sputum samples were collected from all participants for tuberculosis diagnostic tests (smear microscopy, GeneXpert, MGIT culture). Blood and urine samples were collected from HIV-positive women for cluster-of-differentiation-4 cell count, interferon gamma release assay and tuberculosis lateral flow urine lipoarabinomannan tests.

**Results:**

We enrolled 990 women; 52% were pregnant and 47% were HIV-positive. The prevalence of tuberculosis among HIV-positive pregnant women was 5% (95% confidence interval [CI]: 2–7) and among postpartum women it was 1% (95%CI: -1–3). Tuberculosis prevalence was 2% (95%CI: 0–3) in HIV-negative pregnant women and 1% (95%CI: -1–2) in HIV-negative postpartum women. The national tuberculosis symptom screening tool failed to identify women who tested tuberculosis-culture positive.

**Conclusion:**

Routine tuberculosis symptom screening alone is insufficient to rule out tuberculosis in pregnant and postpartum women. Only sputum culture maximised the detection of tuberculosis, indicating a need to balance access and cost in developing countries.

## Introduction

There has been a major decline in maternal mortality over the past two decades.^1^ However, pregnancy poses several challenges to tuberculosis management because of its adverse effects on diagnosis and treatment outcomes.^2,3,4^ The exact burden of tuberculosis among pregnant women is undefined.^5^ The incidence of tuberculosis among postpartum women is unknown and difficulties with diagnosis suggest underestimation.^6^ In 2015, the World Health Organization (WHO) advocated for research on new diagnostic methods targeting pregnant and postpartum women including HIV-positive women^7^ and inexpensive tuberculosis screening algorithms for this population.^8^

The clinical presentation of tuberculosis may be similar to some manifestations of pregnancy, making tuberculosis diagnosis in this population difficult.^6^ The presence of tuberculosis during pregnancy may result in a threefold increase in adverse birth outcomes such as preterm birth, low weight at birth and foetal growth restriction.^8,9^ Screening for tuberculosis in women of reproductive age is imperative, because concomitant tuberculosis disease causes higher case fatality rates than in men of the same age.^10^

Active tuberculosis disease, especially when treated late or left untreated, is likely to result in severe adverse outcomes affecting both mother and baby,^11,12,13^ with an estimated 3.4-fold increase in infant mortality.^14,15^ The WHO has recommended three options for tuberculosis symptom screening based on availability of Xpert MTB/RIF assay, chest X-ray and also HIV status of the individual.^16^ The option one algorithm uses a cough lasting more than 2 weeks to screen positive, option two uses any tuberculosis symptom and option three relies on positive chest X-ray findings.^16^ Option two of the WHO symptom-screening algorithm gives a positive screen if a cough of any duration or any other tuberculosis symptom is evaluated.^16^ The national tuberculosis symptom screening tool (NTBSS) adapted option one of the WHO screening algorithm, through which individuals screen positive if there is a cough of at least 2 weeks duration,^16^ or a cough plus any other symptom of fever or unexplained loss of weight or night sweats, or if any two symptoms are present.^16,17^ Despite developments in screening and diagnosis, emerging data show that the WHO-recommended four-symptom screen may miss persons with tuberculosis disease.^4^ Studies on HIV-positive pregnant women found that the sensitivity of any one of the four tuberculosis symptoms was 28% in South Africa^18^ and 42.9% in Kenya.^19^

Few studies have considered the potential use of tuberculosis lateral flow urine lipoarabinomannan (LF-LAM) as an add-on to the tuberculosis screening algorithm for HIV-positive pregnant and postpartum women.^20^ Xpert^®^ MTB/RIF testing has been rolled out in Eswatini and is the preferred tuberculosis diagnostic method for women. However, a study among HIV-positive pregnant women in Kenya reported Xpert^®^ MTB/RIF sensitivity of 43% and a specificity of 100%, when compared to *Mycobacterium tuberculosis* culture results^19^ as the gold standard. Bactec MGIT 960 liquid culture has been shown to have a sensitivity and specificity of 100% and 93.3%.^21^

Tests for latent tuberculosis infection have shown mixed value for determining the presence of the infection.^22^ Existing tests for latent tuberculosis infection include the tuberculin skin test (TST) and the newer interferon-gamma release assays (IGRAs) but both have drawbacks.^23,24^ The limitations of the TST include low sensitivity and specificity among HIV-positive patients and possibly among pregnant women.^11^ Similarly, a study in Kenya using the T-SPOT TB IGRA showed a more than threefold increased risk of active tuberculosis or mortality among pregnant women who tested positive using IGRA.^25^

In Eswatini, tuberculosis prevalence among pregnant women is not documented. Use of the recommended WHO four-symptom screen has identified very few pregnant women with positive results.^26^ The objectives of this study were to determine the prevalence of bacteriologically confirmed tuberculosis among the study population of HIV-positive and HIV-negative pregnant and postpartum women and to identify effective tuberculosis screening algorithms.

## Methods

### Ethical considerations

Ethical approval was obtained from the Eswatini National Health Research Board (formerly Scientific and Ethics Committee [Approval reference: MH/599C]), CDC Institutional Review Board (IRB) (Reference: CGH-HSR Tracking #: 2015-196), and University Research Co. LLC IRB (02 March 2015). Participants signed informed consent written in their preferred language (SiSwati or English). Participants received no incentives but were reimbursed transport costs for additional visits to read TST. Diagnostics tests and treatment, if required, were free of out-of-pocket charges.

### Study design

We conducted a cross-sectional study enrolling pregnant and postpartum women, aged 18 years and older, attending antenatal and postnatal care clinics, from 01 April to 30 November 2015 at three public health facilities in three of the four regions of Eswatini. Sociodemographic and clinical data, including past tuberculosis screening results where applicable, were collected. Eswatini is categorised by WHO as a high tuberculosis/HIV burdened country with a co-infection rate of 70% and a tuberculosis incidence rate of 398 per 100 000 population.^27^

### Inclusion criteria

Participants were pregnant and postpartum women who were not on anti-tuberculosis treatment at enrolment or who had not taken anti-tuberculosis medicines, including isoniazid for tuberculosis preventive therapy, within the 2 months preceding enrolment, based on documented evidence from patient clinical records, and who provided informed consent. Four groups of women were enrolled: HIV-positive pregnant, HIV-negative pregnant, HIV-positive postpartum, and HIV-negative postpartum.

### Study population and sample size

A sample size of 183 in each group was determined and a full narrative of sample size calculation is fully described in our protocol paper titled ‘Screening in Maternity to Ascertain Tuberculosis Status (SMATS) study’.^4^ Participants were consecutively enrolled until the sample size was reached.

### Clinical and laboratory procedures

#### Symptom screening

All participants were screened using the WHO-recommended national tuberculosis four-symptom screening (standard NTBSS) tool. Participants held clinic cards which were checked for evidence of tuberculosis symptom screening at their last clinical encounter to ascertain routine tuberculosis screening coverage at their previous visit. Participants were screened as positive using the standard NTBSS tool, if they had a cough lasting at least 2 weeks,^16^ or a cough lasting less than 2 weeks plus any other symptom of fever or unexplained loss of weight or night sweats, or if any two symptoms were present.^16,17^ Enhancements of the tuberculosis screening tool were done by adding to the four symptom screening tool any history of contact with a person on tuberculosis treatment or who had been diagnosed with tuberculosis and the presence of tuberculosis symptoms within the household inhabitants. We measured sensitivity, specificity and predictive values (positive and negative) of the WHO-recommended four symptom tuberculosis screening tool among HIV-positive and HIV-negative pregnant and postpartum women compared with sputum culture, the gold standard for *M. tuberculosis* detection.

#### Radiological procedures

Even though chest radiographs are not contraindicated in pregnancy, chest radiographs were only carried out among postpartum women (both HIV-positive and HIV-negative) to eliminate risk of radiation exposure to the foetus.

#### Specimen collection and laboratory procedures

Two samples of sputum (for Xpert^®^ MTB/RIF, smear microscopy and culture using BACTEC TM MGIT 960) were collected from all participants.^4^ Sputum samples were collected through the production of spontaneous self-expectorated phlegm (preferred method), sputum induction through nebulising with hypertonic saline or, if both the above failed, the participant received a sputum container to take home and attempt to produce an early morning sputum sample and bring it back to the health facility. Testing of sputum samples was done at the National Tuberculosis Reference Laboratory in Mbabane. Tuberculosis culture was the gold standard test and in situations where sputum samples were insufficient for the three tests, culture was prioritised ahead of Xpert^®^ MTB/RIF and smear microscopy. A urine sample for the LF-LAM test and two 4 mL blood samples for IGRA testing and cluster-of-differentiation-4 (CD4) cell count testing were collected. Interferon-gamma release assays and LF-LAM were only done for HIV-positive women due to limited evidence on IGRA use^28^ and existing WHO recommendations on LF-LAM use in HIV-positive individuals.^29^ TST was also done and the induration was read after 48 hours – 72 hours. IGRA and TST procedures were explained to all participants and only HIV-positive participants were then asked which test they preferred between IGRA and TST.

Sample collection and storage followed national standard operating procedures for urine and blood collection and manufacturer’s instructions for the Determine^TM^ Tuberculosis LF-LAM test (Abbott Laboratories, Lake Bluff, Illinois, United States) and IGRA testing.^4^ All sputum specimens were analysed by means of: (1) Xpert^®^ MTB/RIF assay (Cepheid, Sunnyvale, California, United States), (2) concentrated Ziehl-Neelsen microscopy, (3) liquid–medium culture method (BACTEC^TM^ MGIT 960^TM^ TB Diagnostic System; Becton, Dickinson & Company [BD], Crystal Lake, New Jersey, United States), and (4) MGIT 960^TM^ DST (BD, Crystal Lake, New Jersey, United States). Positive cultures were identified as *M. tuberculosis* using the tuberculosis Ag MPT64 Rapid^®^ assay (Standard Diagnostics, Inc., Yongin, South Korea).^30^ Interferon-gamma release assays and CD4 cell count testing were done at Lancet Laboratory in Johannesburg, South Africa. IGRA blood specimens were collected directly into IGRA tubes used for QuantiFERON-TB Gold in-Tube assay (Cellestis Ltd, Carnegie, Victoria, Australia).^31,32^ CD4 cell count tests were conducted by BD FACSCalibur™ flow cytometry (BD Biosciences, San Jose, California, United States) using venous blood collected in sterile four millilitre BD Vacutainer EDTA tubes by trained study nurses.

### Data management and data analysis

#### Data collection

The data collection tools were matched to the tools used for routine data collection at health facilities. Demographic fields in the data collection forms were adapted from client cards. Data fields for tuberculosis symptom screening were adapted from the national tuberculosis screening tool. Patient information was anonymised.

#### Data analysis

**FIGURE 1:**
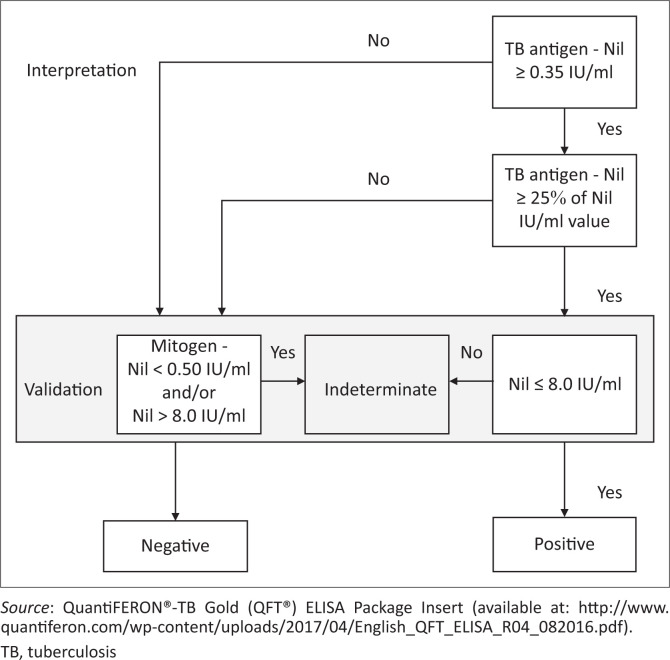
Interpretation of interferon-gamma release assays results.

Data were entered in Epi Info™ (Centers for Disease Control and Prevention, Atlanta, Georgia, United States) and Research Electronic Data Capture (REDCap; Vanderbilt University, Nashville, Tennessee, United States), and data extraction tools were cross-checked to validate conflicting fields. Laboratory results were compared with the electronic study results file generated from the laboratory, and participant identity numbers were used to relate the data. We evaluated both option one and option two of the WHO four symptom screening algorithms. TST numeric readings were recoded as positive, if the length of the induration was ≥ 5 mm for HIV-positive participants or ≥ 10 mm, if the participant was HIV-negative.^32,33^ All other lengths, including 0 mm, were recoded as negative according to existing literature and CDC guidance on interpretation of TST results.^32,33^ IGRA was done at a private laboratory according to manufacturer’s recommendations and the differences in readings between QuantiFERON-tuberculosis Gold in-Tube tuberculosis antigen, tuberculosis nil and tuberculosis mitogen were used to interpret positive, negative and indeterminate results, respectively (Figure 1).^34^

Frequencies and proportions were used to describe participant characteristics and related clinical data. Diagnostic parameters of sensitivity, specificity and positive and negative predictive values analyses for tuberculosis symptoms, and tuberculosis diagnostic tests were calculated in STATA version 13 (© 1985–2013 StataCorp LLC, College Station, Texas, United States). Using logistic regression, associations between culture-positive tuberculosis and HIV status and pregnancy or postpartum status variables were determined. Other sociodemographic and clinical factors were considered for inclusion in the multivariate model if the *p*-value was ≤ 0.1 on bivariate analysis. Factors that perfectly predicted the outcome were excluded. Estimates were reported with 95% confidence intervals (CIs) and corresponding *p*-values.

## Results

### Description of study participants

**FIGURE 2:**
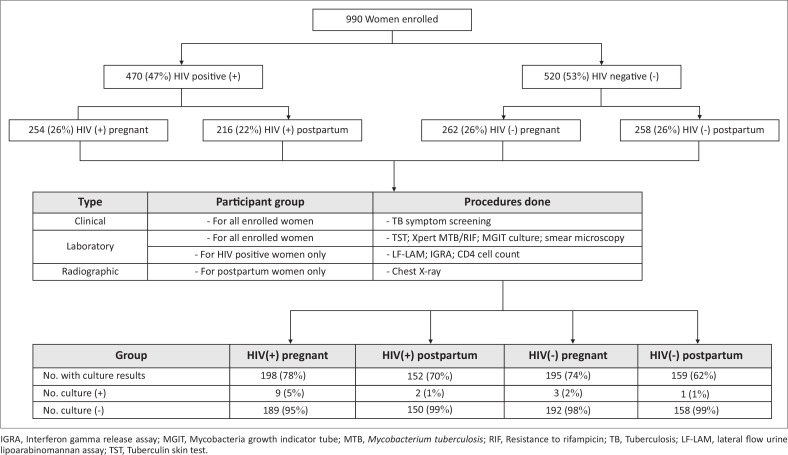
Enrolment categories and culture results of pregnant and postpartum women attending antenatal and postnatal care clinics in Eswatini between April and November 2015.

Of the 990 women who were enrolled, 516 (52%) were pregnant: 101 (10%) in first trimester, 219 (22%) in second trimester and 196 (20%) in third trimester (Figure 2). The remaining 474 (48%) were all postpartum women. Among the participants, 470 (47%) were HIV-positive and, among them, 434 (92%) were on antiretroviral therapy.

Most women (790, 80%) had secondary or tertiary education, and 300 (30%) were in formal employment (Table 1). The median household density (number of people per room within household) was 1.5 (interquartile range: 1–2 people per room). The median age was 26 (interquartile range: 22–31) years.

**TABLE 1:** Demographic characteristics of pregnant and postpartum women attending antenatal and postnatal care clinics in Eswatini between April and November 2015.

Characteristic	All participants (*N* = 990)				Participants with valid culture results (*n* = 704)								Chi^2^†
					TB culture positive				TB culture negative				
	*n*	%	median	IQR	*n*	%	median	IQR	*n*	%	median	IQR	
Age (median)†	26	-	-	22–31	26	-	-	22–31	26	-	-	22–32	0.932
**Participant category**													0.057
Pregnant	516	52	-	-	12	80	-	-	381	55	-	-	
Postpartum	474	48	-	-	3	20	-	-	308	45	-	-	
**Screened for TB symptoms at last routine clinic visit**													0.760
Yes	716	72	-	-	12	80	-	-	528	77	-	-	
No	274	28	-	-	3	20	-	-	161	23	-	-	
**Region of residence**													0.001
Northern	355	36	-	-	12	80	-	-	232	34	-	-	
Central	343	35	-	-	1	7	-	-	254	37	-	-	
Southern	292	29	-	-	2	13	-	-	203	29	-	-	
**Education level**													
High school/tertiary level	790	80	-	-	11	73	-	-	546	79	-	-	0.577
Primary school level	200	20	-	-	4	27	-	-	143	21	-	-	
**Employment status**				-			-	-					
Employed	300	30	-	-	5	33	-	-	217	31	-	-	0.880
Unemployed	690	70	-	-	10	67	-	-	472	69	-	-	-
**Median household density† (number of people/room)**	-	-	1.5	[1–2]	2	-	-	0.8–2.7	-	-	1.5	1–2	0.704
**HIV status**													0.064
Negative	520	53	-	-	4	27	-	-	350	51	-	-	
Positive	470	47	-	-	11	73	-	-	339	49	-	-
**CD4 count (cells/ml)**‡													
< 200	38	8	-	-	0	0	-	-	28	9	-	-	0.358
≥ 200	412	92	-	-	9	100	-	-	298	91	-	-	-
**On ART**‡													0.350
Yes	434	92	-	-	11	100	-	-	314	93	-	-	
No	36	8	-	-	0	0	-	-	25	7	-	-	
**Cough present**													0.534
Yes	103	10	-	-	1	7	-	-	82	12	-	-	
No	887	90	-	-	14	93	-	-	607	88	-	-	
**Fever present**													0.482
Yes	29	3	-	-	0	0	-	-	22	3	-	-	
No	961	97	-	-	15	100	-	-	667	97	-	-	
**Weight loss present**													0.385
Yes	48	5	-	-	0	0	-	-	33	5	-	-	
No	942	95	-	-	15	100	-	-	656	95	-	-	
**Night sweats present**													0.401
Yes	38	4	-	-	0	0	-	-	31	5	-	-	
No	952	96	-	-	15	100	-	-	658	95	-	-	
**At least one TB symptom**													0.198
Yes	181	18	-	-	1	7	-	-	138	20	-	-	
No	809	82	-	-	14	93	-	-	551	80	-	-	

ART, antiretroviral therapy; CD4, cluster of differentiation 4; IQR, interquartile range; TB, tuberculosis.

† , Kruskal Wallis test used for medians;

‡ , for HIV-positive participants only.

### Tuberculosis screening and sputum collection

Of the 990 participants screened, 48 (5%) screened positive for tuberculosis using the NTBSS tool. There were 181 (18%) with at least one tuberculosis symptom (Table 1). Participants who screened positive for specific tuberculosis symptoms included 103 (10%) with a cough of any duration, 38 (4%) with night sweats, 29 (3%) with fever and 48 (5%) with weight loss. Among 516 pregnant women, 433 (84%) were screened for tuberculosis at their last clinical encounter compared with 283 (60%) of the 474 postpartum women. In 531 (54%) patients, sputum collection was spontaneous, 158 (16%) by induction and 87 (9%) as early morning samples. However, 214 (22%) participants failed to produce sputum using any of the three methods. In total, 776 (78%) participants produced sputum samples and 758 (98%) had samples available for culture testing and 704 (93%) had valid culture results. However, only 361 (47%) had sputum samples available for Xpert^®^ MTB/RIF testing and Xpert^®^ MTB/RIF results were available for 361 (100%) participants, although 18 (5%) of those with Xpert^®^ MTB/RIF results had no culture results for comparison.

### Prevalence of tuberculosis

Tuberculosis status was either bacteriologically confirmed or ruled out by culture testing in 704 (93%) participants who had sputum samples available for culture. The overall prevalence of tuberculosis in this cohort of pregnant and postpartum women was 2% (95% CI: 1–3) (Table 2). *M. tuberculosis* was found in 15 (2%) participants and 12 (80%) were pregnant. The prevalence of tuberculosis among pregnant women was 3% (95% CI: 1–5) compared to 1% (95% CI: 0–2) in postpartum women. The prevalence of tuberculosis among those who were HIV-negative was 1% (95% CI: 0–2) compared to a prevalence of 3% (95% CI: 1–5) among those who were HIV-positive (Table 2).

**TABLE 2:** Prevalence of tuberculosis, by HIV status, among pregnant and postpartum women attending antenatal and postnatal care clinics in Eswatini between April and November 2015.

Patient category	Number of participants	No. with culture results		TB culture positive	Prevalence		Pooled prevalence	
		*n*	%		%	95% CI	%	95% CI
**HIV-positive**							3	1–5
Pregnant	254	198	78	9	5	2–7		
Postpartum	216	152	70	2	1	−1–3		
**HIV-negative**							1	0–2
Pregnant	262	195	74	3	2	0–3		
Postpartum	258	159	62	1	1	−1–5		
**Total (HIV-positive and HIV-negative)**							2	1–3
Pregnant	516	393	76	12	3	1–5		
Postpartum	474	311	66	3	1	0–2		

CI, confidence interval; TB, tuberculosis.

### Acceptability of tuberculosis skin test and interferon-gamma release assay

A total of 450 participants chose between TST and IGRA as their preferred test method. Of this number, 277 (62%) chose TST as the preferred method compared to 173 (38%) for IGRA (data not shown in tables). Among the 540 with no decision on preferred method, 512 (95%) were not sure, and 28 (5%) chose not to respond to the question. Tuberculin skin tests were done on 961 (97%) participants, of which 659 (69%) came back for reading within 48 h – 78 h, whereas 302 (31%) were lost to follow-up. Of the 659 participants who had TST results, 237 (36%) were TST-positive. IGRAs were done on 465 HIV-positive participants. Of the 465 IGRA results, 153 (33%) were positive, 273 (59%) negative, 23 (5%) indeterminate and 16 (3%) were missing.

### Performance of tuberculosis screening and diagnostic tests

The standard NTBSS tool failed to identify women with tuberculosis disease with a sensitivity of 0% (95% CI: 0–29) among HIV-positive and 0% (90% CI: 0–60) among HIV-negative participants (Table 3). The enhanced screening tool, by including history of contact with a person with tuberculosis or presence of tuberculosis symptoms within the household of the participant, did not improve the sensitivity of the standard NTBSS tool. Only the inclusion of a tuberculosis contact history significantly improved the sensitivity of the algorithm to 18% among HIV-positive women, although specificity decreased from 94% to 62%.

**TABLE 3:** Sensitivity, specificity, positive and negative predictive values of tuberculosis screening symptoms and diagnostic tests in pregnant and postpartum women attending antenatal and postnatal care clinics in Eswatini between April and November 2015.

Test	HIV positive									HIV negative								
	*n*	SN		SP		PPV		NPV		*n*	SN		SP		PPV		NPV	
		%	95% CI	%	95% CI	%	95% CI	%	95% CI		%	95% CI	%	95% CI	%	95% CI	%	95% CI
**‘Standard NTBSS tool’**- WHO screening Option #1	350	0	0–29	94	91–97	0	0–18	97	94–98	354	0	0–60	94	91–97	0	0–17	99	97–100
**‘At least one TB symptom’**- WHO screening Option #2	350	9	0.2–47	84	79–87	2	0.0–9	97	94–98	354	0	0–60	77	72–81	1	0–4	99	96–100
Household TB symptoms	350	9	0.2–41	95	92–97	6	0.1–29	97	95–99	354	0	0–60	90	86–93	0	0–10	99	97–100
**Enhanced**: ‘NTBSS tool’ + household TB symptoms	350	9	0.2–41	90	86–93	3	0.1–15	97	94–99	354	0	0–60	86	82–89	0	0–7	99	98–100
**Enhanced**: ‘NTBSS tool’ + household TB symptoms+ TB contact	350	18	2–52	62	57–64	2	0.2–5	96	92–98	354	0	0–60	57	52–62	0	0–2	98	95–100
**Enhanced**: At least one symptom plus TB contact history	350	18	2–52	64	59–69	2	0.2–6	96	93–98	354	0	0–60	59	53–64	0	0–3	98	95–100
Chest X-Ray†	143	50	1–99	88	81–93	6	0.1–27	99	96–100	140	‡	‡	‡	‡	‡	‡	‡	‡
Xpert® MTB/RIF	175	0	0–60	99	96–100	0	0–84	98	94–99	-	§	§	§	§	§	§	§	§
LF-LAM	327	11	0–48	94	91–96	5	0–25	97	95–99	-	-	-	-	-	-	-	-	-
LF-LAM (if CD4 count ≤ 100)	28	All 6 participants with CD4 count ≤ 100 tested negative with LF-LAM.								-	-	-	-	-	-	-	-	-
TST	255	20	3–56	67	61–73	2	0.3–8	95	-	294	0	0–84	59	53–65	0	0–3	99	96-100
IGRA	319	22	3–60	63	57–68	2	0.2–6	97	-	-	-	-	-	-	-	-	-	-

CD4, cluster of differentiation 4; NPV, negative predictive value; CI, confidence interval; NTBSS, National TB symptom screening tool; PPV, positive predictive value; IGRA, interferon gamma release assay; SN, sensitivity; IQR, interquartile range; SP, specificity; LF-LAM, lateral flow urine lipoarabinomannan; TB, tuberculosis; TST, tuberculin skin test; WHO, World Health Organization.

† , among postpartum women only.

‡ , All cultures were negative in this group.

§ , All Xpert® tests were negative in this group.

*M. tuberculosis* was detected in two (1%) of the 361 Xpert MTB/RIF results, and no rifampicin resistance was detected in either one. No *M. tuberculosis* was detected in the remaining 359 (99%) samples. Sputum smear microscopy was done on 724 (73%) participants, and 4 (1%) had acid-alcohol-fast bacilli. LF-LAM was done on 411 (87%) participants and 327 (80%) had culture results. When compared to culture, the sensitivity of both the tuberculosis screening and the tuberculosis diagnostic tests were less than 50% in both HIV-positive and HIV-negative women (Table 3).

### Association between socio-demographic and clinical covariates and tuberculosis culture diagnostic algorithm

Specific algorithms could not be analysed due to the missing data of tests used to construct the different algorithms, as well as low prevalence of tuberculosis symptoms among our study participants. The study showed that those who were HIV-positive had a threefold risk of culture-positive tuberculosis compared to HIV-negative individuals (odds ratio = 3.23; 95% CI: 1.00–10.40, *p* = 0.05) (Table 4). Those residing in the Central region (odds ratio = 0.08; 95% CI: 0.01–0.61, *p* = 0.015) and Southern region (odds radio = 0.19; 95% CI: 0.04–0.89, *p* = 0.035) were independently less likely to have culture-positive tuberculosis compared to the northern region (reference region) and (Table 4).

**TABLE 4:** Factors associated with culture positive tuberculosis in pregnant and postpartum women attending antenatal and postnatal care clinics in Eswatini between April and November 2015.

Variable	Univariate			Multivariate		
	Odds ratio	95% CI	*p*	Odds ratio	95% CI	*p*
Age	0.99	0.91–1.08	0.873	Omitted	-	-
**HIV status**						
Negative	1	Ref	-	1	Ref	-
Positive	2.84	0.90–9.00	0.076	3.23	1.00–10.40	0.050
**Category**						
Pregnant	1	Ref	-	1	Ref	-
Postpartum	0.31	0.09–1.11	0.071	0.43	0.12–1.58	0.204
**Region of residence**						
Northern	1	Ref	-	1	Ref	-
Central	0.08	0.01–0.59	0.014	0.08	0.01–0.61	0.015
Southern	0.19	0.04–0.86	0.031	0.19	0.04–0.89	0.035

CI, confidence interval; Ref, reference category.

## Discussion

### Summary of key findings

According to our review, this is a unique study in this setting to determine the burden of tuberculosis among pregnant and postpartum women regardless of the presence of tuberculosis symptoms. We observed that higher proportions of pregnant women (84%) were previously screened for tuberculosis during their last clinic visit prior to enrolment compared to postpartum women (60%). However, these were lower than the universal screening (99%) reported among people living with HIV attending antiretroviral therapy clinics in Eswatini.^35^

Even though 80% of participants who were confirmed to have tuberculosis disease were pregnant, there were no statistical differences between the prevalence of tuberculosis in pregnant and postpartum women. The highest prevalence of tuberculosis was among HIV-positive pregnant women (5%), which is comparable to the 3.3% observed in neighbouring South Africa.^11^ Although 93% of participants who were found to have active tuberculosis reported no tuberculosis symptoms, there were no differences in tuberculosis prevalence between those reporting symptoms and those with no symptoms. A study conducted in South Africa found a higher tuberculosis prevalence among patients who did not report symptoms of tuberculosis.^18^ A study from Ethiopia did not find any person with active tuberculosis disease among pregnant women but did not test for tuberculosis among those who did not have tuberculosis symptoms.^36^ This has significant public health implications for tuberculosis control, considering previous reports that asymptomatic patients with culture-positive tuberculosis can transmit tuberculosis.^18^

The WHO four-symptom NTBSS screening tool failed to identify women with active tuberculosis disease, as the majority of women with confirmed tuberculosis disease did not have symptoms of tuberculosis. Almost a third of participants who had a TST done did not have results, because participants did not come back within the stipulated time for reading, despite the provision of transport imbursements for additional visits for TST reading.

Pregnancy is known to suppress the T1-helper pro-inflammatory response, resulting in masking of tuberculosis symptoms and increased susceptibility to *M. tuberculosis* reactivation^8^ and primary infection with *M. tuberculosis*.^37^ Women are unlikely to show typical symptoms like sweating at night and fever, and these are further masked by pregnancy.^8,17^ Weight loss can be masked by physiological changes during pregnancy.^18^ The low sensitivity of the tuberculosis screening tool has been reported in many other studies.^18,19,38,39^ Adapting the screening tool to include a history of tuberculosis contact as an independent indicator of positive tuberculosis screen improved sensitivity by 18% but only in HIV-positive women. This sensitivity (18%) is still too low for effective tuberculosis case finding and ruling out tuberculosis among pregnant and postpartum women. LaCourse et al.^19^ also showed poor performance of the four-symptom screening tool in HIV-positive pregnant women in Kenya. Tuberculosis diagnosis in pregnancy is often delayed due to atypical symptoms.^11,39,40^ Culture prevailed as the reliable gold standard to diagnose tuberculosis; some authors recommend that it should be mandatory for establishing tuberculosis diagnosis in this group.^11,39^

A false-negative symptom screening, which is the initial screening method for triaging for tuberculosis diagnostic testing, will often lead to a delay in diagnosis and treatment of active tuberculosis and consequently poor foetal and maternal outcomes.^17^ A false-positive screening result leads to inconvenient and costly laboratory procedures.^17^ However, the routine symptom screening tool had high negative predictive values (97% – 99%), which supports its utility in identifying people who are unlikely to have tuberculosis, especially people living with HIV. Therefore, those who screen negative with the standard NTBSS tool and are at high risk of progressing from latent tuberculosis to active tuberculosis can be given tuberculosis preventive therapy in this setting of high HIV-tuberculosis burden.^39,41^

We demonstrated that TST had poor performance when used as a screening method for exposure to tuberculosis and its feasibility is further challenged by a third of participants who were lost to follow-up for a reading of skin reaction. However, alternative follow-up methods, including home visits, should be included to complete the TST readings. IGRA was a less preferred screening method compared to TST, possibly due to the need for blood draw. In addition, IGRA demands laboratory infrastructure^42^ compared to TST and is currently available only in private laboratories in this setting.

### Strengths and limitations

We considered it a strength that our methodology included participants who did not have the usual symptoms of tuberculosis, minimising the risk of under-reporting of true tuberculosis prevalence in this study population.^39,43^ We also induced sputum in women unable to produce sputum spontaneously, thus maximising the tuberculosis diagnostic yield.^19^ In addition to symptom screening, we attempted five tuberculosis tests for all participants.

We had planned to test 10 different algorithms using different testing combinations and ordering of individual diagnostic tests. However, given the low agreement, the low number of positives, and the poor performance of the WHO symptom screening tool (several of the algorithms began with the WHO symptom screening), the analysis was of no utility, and we did not include these results in this report. Although unintended by the study design, more than half of the participants did not have an Xpert^®^ MTB/RIF assay done, which is a near point-of-care test that allows results to be quickly available (as early as 2 h) to clients and service providers, leading to a quick clinical management decision, unlike culture which may take several weeks.

### Conclusion

The four-symptom screening tool appears likely to miss women with active tuberculosis. Without sensitive, symptom-based tuberculosis screening in this subpopulation, a high index of suspicion of tuberculosis is necessary and factors such as a history of tuberculosis contact should prompt clinicians to consider tuberculosis in their differential diagnosis, especially in a setting of high HIV- tuberculosis burden. Bold, deliberate decisions to invest in laboratory-based, quality-assured culture testing are required to maximise detection of people who have active tuberculosis disease in countries with a high HIV- tuberculosis burden in order to end tuberculosis by 2035 as envisaged by the WHO’s End TB Strategy.^44^ However, the feasibility of increasing access to culture in this setting is confronted by costs related to culture testing, transportation, specimen storage and lengthy waiting periods for culture results by clinicians and patients. Therefore, low-income and middle-income countries should strike the right balance to ensure access to culture for those who could benefit from culture and availability of the newer, more sensitive Xpert^®^ MTB/RIF Ultra platform for rapid tuberculosis diagnosis.

Prevalence of tuberculosis was particularly high (4.5%) among HIV-positive pregnant women and low (0.6%) among HIV-negative postpartum women. Although we were unable to test different tuberculosis screening algorithms, the poor performance of the standard NTBSS tool that serves as the entry to tuberculosis services highlights the challenge of diagnosing tuberculosis in pregnant and postpartum women.
